# SCAPS study on the effect of various hole transport layer on highly efficient 31.86% eco-friendly CZTS based solar cell

**DOI:** 10.1038/s41598-023-44845-6

**Published:** 2023-10-27

**Authors:** Rahutosh Ranjan, Nikhil Anand, Manish Nath Tripathi, Neelabh Srivastava, Arvind Kumar Sharma, Masamichi Yoshimura, Li Chang, Rajanish N. Tiwari

**Affiliations:** 1 Department of Physics, School of Physical Sciences, Mahatma Gandhi Central University, Motihari, India; 2 Department of Chemistry, School of Physical Sciences, Mahatma Gandhi Central University, Motihari, India; 3 Institute of Science, Banaras Hindu University, Varanasi, 221005 Uttar Pradesh India; 4 Toyota Technological Institute, 2-12-1 Hisakata, Tampaku-Ku, Nagoya, 468-8511 Japan; 5 Department of Materials Science and Engineering, National Yang Ming Chiao Tung University, Hsinchu, Taiwan

**Keywords:** Chemistry, Materials science, Mathematics and computing, Physics

## Abstract

Copper Zinc Tin Sulphide (CZTS) is a propitious semiconductor for active absorber material in thin-film solar cells (SCs). Here, SC architecture comprising FTO/ZnS/CZTS/variable HTLs/Au is discussed. Fluorine-doped tin oxide (FTO) and gold (Au) are used as front and back contacts, respectively. Zinc sulphide (ZnS) is used as an active electron transport layer (ETL), while different Cu-based materials (Cu_2_O, CuO, CuI, and CuSCN) are used as hole transport layers (HTL). A one-dimensional solar cell capacitance simulator (SCAPS-1D) is utilized to simulate the SC structure. Among different Cu-based HTLs, Cu_2_O is preferred as a potential candidate for high cell performance of CZTS-based SC. The effects of various layer parameters such as thickness, doping density, and carrier concentrations, electron affinity of HTL and absorber, respectively, are also discussed. After optimization of the device, variation of operating temperature and the effect of series and shunt resistance are also taken into consideration. The optimized results of thickness and acceptor concentration (N_A_) of absorber material are 1.5 µm and approx. 1.0 × 10^19^ cm^−3^, respectively. In addition, the function of HTL (with and without) in the designed SC structure is also studied. Capacitance–voltage (C–V) characteristics are also discussed to get an insight of built-in potential. We have achieved cell performances viz. efficiency = 31.86%, short circuit current density = 32.05 mA/cm^2^, open circuit voltage = 1.19 V, and fill factor = 83.37%.

## Introduction

With the growing population, there has been a great demand for energy, which is mostly fulfilled by using fossil fuels, but solar energy is considered the need of the future due to its various advantages^1^. Fossil fuel is going to deplete one day, but solar energy is entirely renewable and, at no cost, can supply an infinite amount of energy^2^. The dependence on solar energy is growing as a result of new research developments that can enhance its efficiency. In our country, various electrification programmes of the government have been in operation, which highlights the importance of solar power^3^. Among the most essential aspects of any country's advancement and prosperity is energy. When talking about sustainable energy, we have to replace the existing sources with renewable energy supply. The regular and never-ending use of fossil fuels has already led to many problems such as pollution, global warming, health issues, and so on^4^. Therefore, with the ongoing high demand for energy and environmental concerns, researchers have focused on alternative sources (solar energy) that can meet our energy demand and also not contaminate our environment^5^.

CIGS (copper indium gallium (di)selenide) and CdTe SCs dominate the market for thin-film technologies. However, cadmium (Cd) and tellurium (Te) are poisonous^6^ and with the availability of rare elements such as indium (In) and gallium (Ga), the need of the hour is to look for non-toxic and earth-abundant materials that can suitably replace the existing ones with desired characteristics. CZTS is an extrinsic p-type semiconductor known for its ease of preparation due to the availability of its constituent components (copper, zinc, tin, and sulphur)^7–10^. CZTS mainly exist in kesterite form as it is more stable^11,12^. CZTS is ecologically beneficial due to the quantity of harmless elements. Apart from that, CZTS offers strong electrical and optical properties along with excellent stability, making it a more profound material for PV devices^13,14^. CZTS has gained attention across the scientific community due to its promising optoelectronic properties, including high hole mobility, an adjustable bandgap, and earth-abundant elements^15,16^. However, due to poor open circuit voltage (V_oc_), CZTS-based SCs have a deficit in higher conversion efficiency^12^.

Various works have been reported that show that CZTS is the best material for thin-film SCs. It has been observed that the CZTS-based SC with zinc telluride (ZnTe) as a buffer layer revealed an impressive power conversion efficiency (PCE) of 23.47% with the SC structure Mo/CZTS/ZnTe/ZnO/ZnO:Al^17^. In another study, TiO_2_ was used as ETL, and the tin perovskite CH_3_NH_3_SnI_3_ (MASnI_3_) acted as an absorber layer (FTO/TiO_2_/CH_3_NH_3_SnI_3_/CZTS/Au). They obtained short circuit current density (J_SC_) = 31.66 mA/cm^2^, open circuit voltage (V_OC_) = 0.96 V, fill factor (FF) = 67%, and efficiency = 20.28%^18^. Dey et al.^19^ measured SC performance on i-ZnO, InSe, and CZTS simulation structures and found it to be 16.30%. In this structure, CZTS and InSe served as absorber layers and buffer layers, respectively. A new device structure (FTO/ZnO/CdS/CZTS/CZTSe/Mo) is reported by Rana et al. in 2021. In the reported structure, CZTS and CdS were taken as a absorber and buffer layers, respectively. The cell performances were analysed against thickness variations, carrier concentrations, and defect densities (N_t_) and found an overall conversion efficiency of 22.03%^20^. The architecture (MO/CZTS/CdS/ZnO/FTO) was studied by Zyoud et al. in 2021 and has a PCE of 27.72%, including a poor value of V_oc_ of 0.64V^21^. However, Benzetta et al. tried to replace CdS with ZnS and introduced an alternative layer in CZTS-based SC known as a back surface field (BSF), which attains an efficiency of 14.14% and an improved V_oc_ of 0.89 V^12^.

Therefore, the current work focuses on copper-based (p-type) inorganic semiconductors as a viable HTL to address the issue of low V_OC_ and improve the efficiency of CZTS-based SCs. High-hole mobility, high conductivity, great optical transmittance, low cost, and eco-friendliness of copper-based semiconductors enable them to be projected as HTL in SCs^22,23^. Therefore, due to their meritorious properties, CuO, Cu_2_O, CuI, and CuSCN are introduced as different HTLs in our designed SC.

In this study, we have discussed the CZTS-based SC structure comprising FTO/ZnS/CZTS/Cu-based HTLs/Au. In which FTO metal work function (4.4 eV) is used as a front contact, ZnS as an ETL, CZTS as an absorber layer, copper-based materials as HTL, and gold (Au) metal work function (5.1 eV) as a back contact. Generally, CdS is used with CZTS. However, in the present study, CdS is replaced by ZnS due to its toxicity. The effect of thickness variation and acceptor concentrations is calculated for the absorber and HTL. In addition, variations of defect density and electron affinity of the absorber and HTL are studied, respectively. Further, the influence of operating temperature, shunt, and series resistances is well examined for the optimised structure. The capacitance–voltage (C-V) characteristic is also considered to gain an in-depth understanding of junctions. The computed SC performances of the suggested SC structure include an efficiency of 31.86%, FF of 83.37%, J_SC_ of 32.05 mA/cm^2^, and V_OC_ of 1.19 V. In addition, we have also studied the role of HTL (with and without) in the discussed structure.

The commercialization of solar cells could possibly be accelerated by CZTS-based solar cells. CZTS is a non-hazardous, ecologically safe semiconductor that exhibits encouraging PCE but is constrained by its low V_OC_. Therefore, relevant HTMs have been included in numerous studies to boost V_OC_. Thus, this work explores the use of several copper-based HTLs to enhance the efficiency of CZTS-based solar cells. Cu-based HTLs were previously known to be low-cost, non-toxic, highly heat- and chemical-resistant materials with good charge carrier mobility. The improvement in PCE was made possible by optimizing a number of solar cell properties. Cu_2_O is therefore established to be an appropriate HTL for CZTS-based solar cells with good band alignment and an efficiency of 31.86%.

## Materials and methods

### Device design and numerical simulation study

The investigation of SC's photovoltaic (PV) properties through manufacture requires both time and money. Thus, one of the crucial aspects in analysing their PV properties is numerical design and modelling of SC architecture. With the various input material parameters and the modelling process, any solar structure may be designed. They also allow us to study the performance of the SC application by changing the material's characteristics. We can quickly understand the SC device behaviour from the simulation results, which helps us to realize the indicated SC restrictions. Additionally, the software's validity is shown in Table 1 by the fact that SCAPS simulated results closely match with the experimental results.

**Table 1:** Examining several solar cell topologies through simulation and experiment.

Structure	V_oc_ (V)	J_sc_ (mA/cm^2^)	FF (%)	PCE (%)
ITO/ZnO/CdS/CZTS^24^				
Experimental	0.38	13.32	41.92	2.18
Simulated	0.37	13.14	37.9	1.93
Mo/CZTS/CdS/AZO^25^				
Experimental	0.61	17.90	62.00	6.77
Simulated	0.61	17.58	59.45	6.41
(Ni/Al)MgF2/ZnO:AL/i-ZnO/CdS/CZTS/Mo/substrate^26^				
Experimental	0.66	19.5	65.8	8.4
Simulated	0.74	19.5	57.48	8.4

In this study, SCAPS-1D (version 3.3.10), a 1-D simulation of solar cell capacitance, is employed. Prof. Marc Bargeman et al. at the Department of Electronics and Information Systems at the University of Gent in Belgium developed this software^23^. It is utilized in SC modelling, numerical analysis, and the investigation of solar structure characteristics. SCAPS-1D uses stability, Poisson, and semiconductor equations to solve them in order to describe the in-depth analysis of the SC structure^27^. A numerical simulation study of solar cells provides us a better insight of various material properties, which is used during the fabrication of the SCs^4,28^. A thorough study of simulated results enables researchers to sustainable and more efficient way to manufacture low cost and high efficient PV devices^29^.

Table 2 lists the electrical properties of different materials that were employed in this simulation, including the mobility of electrons and holes, relative permittivity, electron affinities, effective density of states (DOSs) in the valence band and conduction band, and acceptor and donor densities^30,31^. The material properties of various Cu-based HTLs are shown in Table 3^32,33^.

**Table 2:** SCAPS simulation parameters for each layer.

Parameter	CZTS^18^	ZnS^30^	FTO^31^
Thickness (µm)	1.5	0.05	0.05
Band gap (eV)	1.4	3.6	3.50
Electron affinity (eV)	4.1	3.9	4.00
Relative dielectric constant	9.0	9.0	9.0
Conduction band effective density of states per cm^3^	2.2 × 10^18^	2.2 × 10^18^	2.2 × 10^18^
Valence band effective density of states per cm^3^	1.8 × 10^18^	1.8 × 10^19^	1.8 × 10^19^
Mobility of e^-^ (cm^2^ s^−1^ V^−1^)	1.0 × 10^2^	1.0 × 10^2^	2.0 × 10^1^
Mobility of hole (e^+^) (cm^2^ s^−1^ V^−1^)	1.25 × 10^1^	1.25 × 10^1^	1.0 × 10^1^
Thermal velocity of e^−^ (cm s^−1^)	1.0 × 10^7^	1.0 × 10^7^	1.0 × 10^7^
Thermal velocity of hole (e^+^) (cm s^−1^)	1.0 × 10^7^	1.0 × 10^7^	1.0 × 10^7^
Shallow uniform donor density (*n*_*D*_) per cm^3^	0	1.1 × 10^18^	1.0 × 10^19^
Shallow uniform acceptor density (*n*_*A*_) per cm^3^	1.0 × 10^19^	0	0
Total defect density (N_t_) per cm^3^	1.0 × 10^15^	1.0 × 10^15^	1.0 × 10^15^

**Table 3:** SCAPS simulation parameters for different HTLs.

Parameter	Cu_2_O^32^	CuO^34^	CuI^32^	CuSCN^33^
Thickness (µm)	0.050	0.050	0.050	0.050
Band gap (eV)	2.17	1.51	3.1	3.4
Electron affinity (eV)	3.2	4.07	2.1	1.9
Dielectric permittivity (relative)	7.11	18.1	6.5	10.0
CB (conduction band) effective density of states (cm^−3^)	2.02 × 10^17^	2.2 × 10^19^	2.8 × 10^19^	2.2 × 10^18^
VB (valence band) effective density of states (cm^−3^)	1.1 × 10^19^	5.5 × 10^20^	1.0 × 10^19^	1.8 × 10^18^
Electron mobility (cm^2^/V s)	2.0 × 10^2^	1.0 × 10^1^	1.0 × 10^2^	1.0 × 10^2^
Hole mobility (cm^2^/V s)	8.0 × 10^1^	1.0 × 10^–1^	4.39 × 10^1^	2.50 × 10^1^
Electron thermal velocity (cm/s)	1.0 × 10^7^	1.0 × 10^7^	1.0 × 10^7^	1.0 × 10^7^
Hole thermal velocity (cm/s)	1.0 × 10^7^	1.0 × 10^7^	1.0 × 10^7^	1.0 × 10^7^
Shallow uniform donor density (N_D_) cm^−3^	0	0	0	0
Shallow uniform acceptor density (N_A_) cm^−3^	1.0 × 10^19^	1.0 × 10^18^	1.0 × 10^18^	1.0 × 10^18^
Total defect density (N_t_) cm^−3^	1.0 × 10^15^	1.0 × 10^15^	1.0 × 10^15^	1.0 × 10^15^

**Figure 1:**
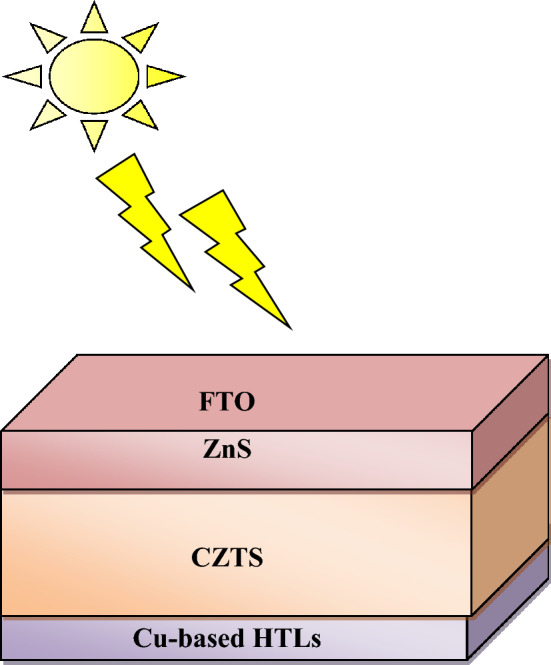
Schematic diagram of simulated CZTS-based solar cell.

**Figure 2:**
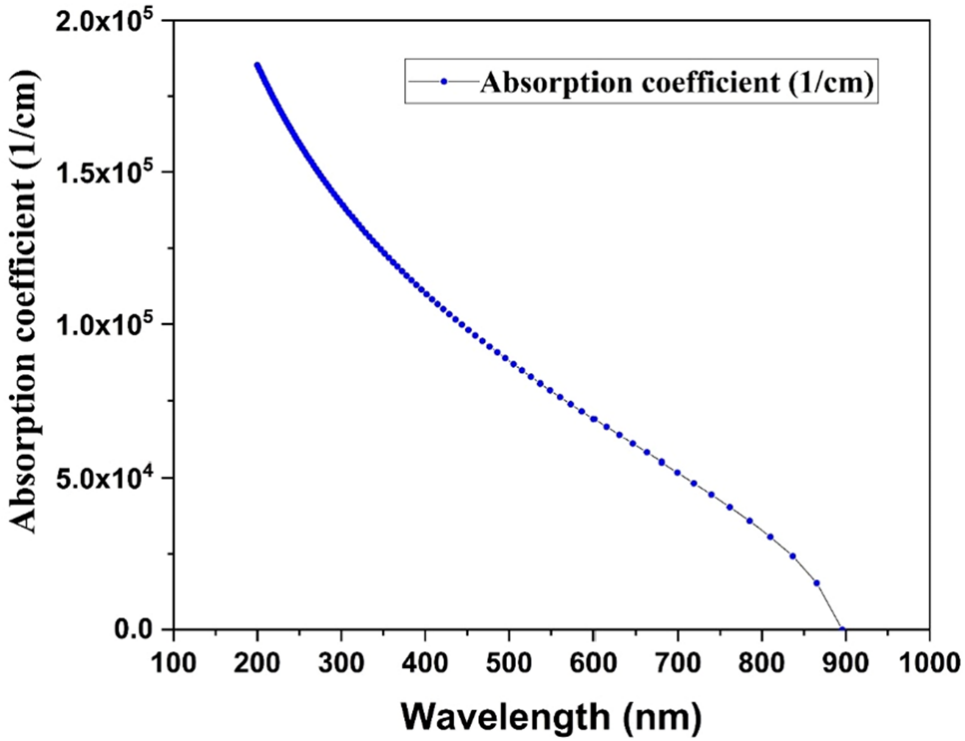
An illustration of absorption coefficient against wavelength of sun spectra for CZTS film.

The designed CZTS-based solar cell structures (FTO/ZnS/CZTS/Cu-based HTLs/Au) is shown in Fig. 1. The simulation is performed at 300 K, and the under AM 1.5 G solar spectrum. It is well known that the absorption coefficient for CZTS absorber layer should be more than 10^4^ cm^−1^. It is clear from Fig. 2 that the absorption coefficient for CZTS is ranged above 10^4^ cm^−1^ which confirmed good an absorption characteristics of absorber material for photovoltaic cell.

## Results and discussion

### Selection of appropriate HTL for SC

**Figure 3:**
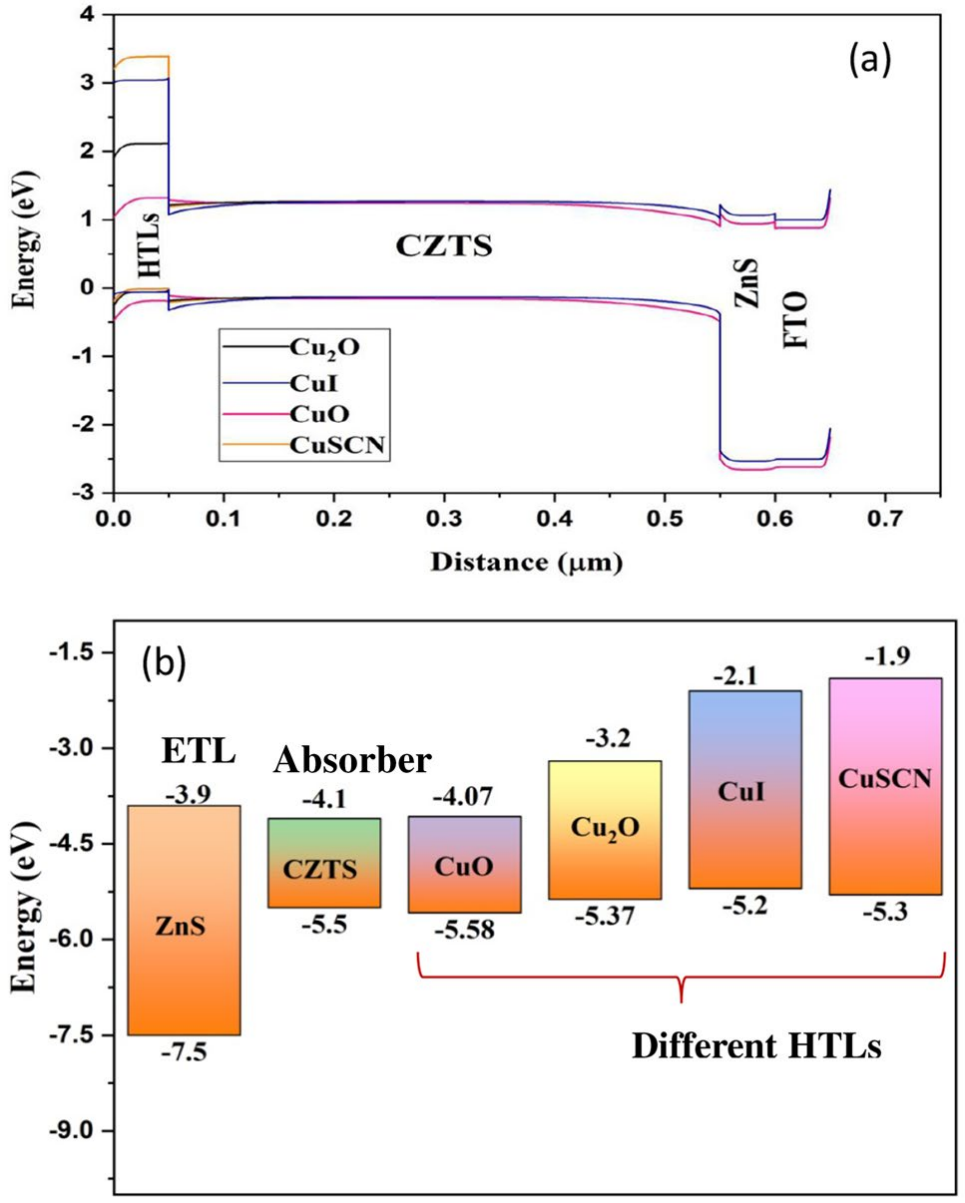
(**a**) Energy band diagram and (**b**) schematic energy band alignment of designed solar cell.

In this study, various Cu-based materials have been proposed as the HTL for a CZTS-based SC. Initially, the simulation process is performed for different HTLs (CuO, Cu_2_O, CuI, and CuSCN). The thickness of different active layers likewise electron transport, various hole transport and absorber materials, are taken to be 50 nm, 50 nm, and 500 nm, correspondingly. Figure 3a and b show the energy band diagram and energy band alignment of the SC using various Cu-based materials as HTL.

As energy band alignment governs the movement of photo-generated charge carriers, which regulates the PV performance of the designed SC^22^. Table 4 shows the calculated valence band offsets (VBO) for each HTL with respect to the CZTS absorber layer, using Eq. (1)^35^;

**Table 4:** The calculated VBO for different HTLs.

HTL	CuO	Cu_2_O	CuI	CuSCN
VBO	+ 0.08	− 0.13	− 0.3	− 0.2

1 $$VBO=\left({{\chi }_{HTL}+{E}}_{{g}_{HTL}}\right)- \left({\chi }_{abs}+{E}_{{g}_{abs}}\right)$$

where, ${\chi }_{HTL}$, ${\chi }_{abs}$ are electron affinities of HTL and absorber layer, respectively. ${E}_{{g}_{HTL}}$, ${E}_{{g}_{abs}}$ are the energy band gap values of HTL and absorber layer, respectively.

From Table 4, it can be observed that the VBO for Cu_2_O, CuI, and CuSCN is negative but the VBO for CuO is positive. A positive VBO means it will form a spike close to the interface of absorber/HTL, which hinders the motion of hole charge carriers^12,36^. However, a negative VBO forms an energy cliff close to the interface of absorber/HTL, which promotes the motion of hole charge carriers. The formation of cliffs and spikes at the absorber/HTL edge alters the efficiency of the designed SC^36^. This can be observed in Table 5, Cu_2_O with a small negative VBO (− 0.13 eV) attains the maximum efficiency with respect to other Cu-based HTLs.

**Table 5:** Performance of CZTS-based SC including various HTLs.

HTL	V_oc_ (V)	J_sc_ (mA/cm^2^)	Fill factor (%)	Efficiency (%)
Cu_2_O	1.030	29.20	83.08	25.01
CuO	0.9109	28.99	82.06	21.68
CuI	1.029	29.16	81.17	24.36
CuSCN	1.029	29.19	82.88	24.91

**Figure 4:**
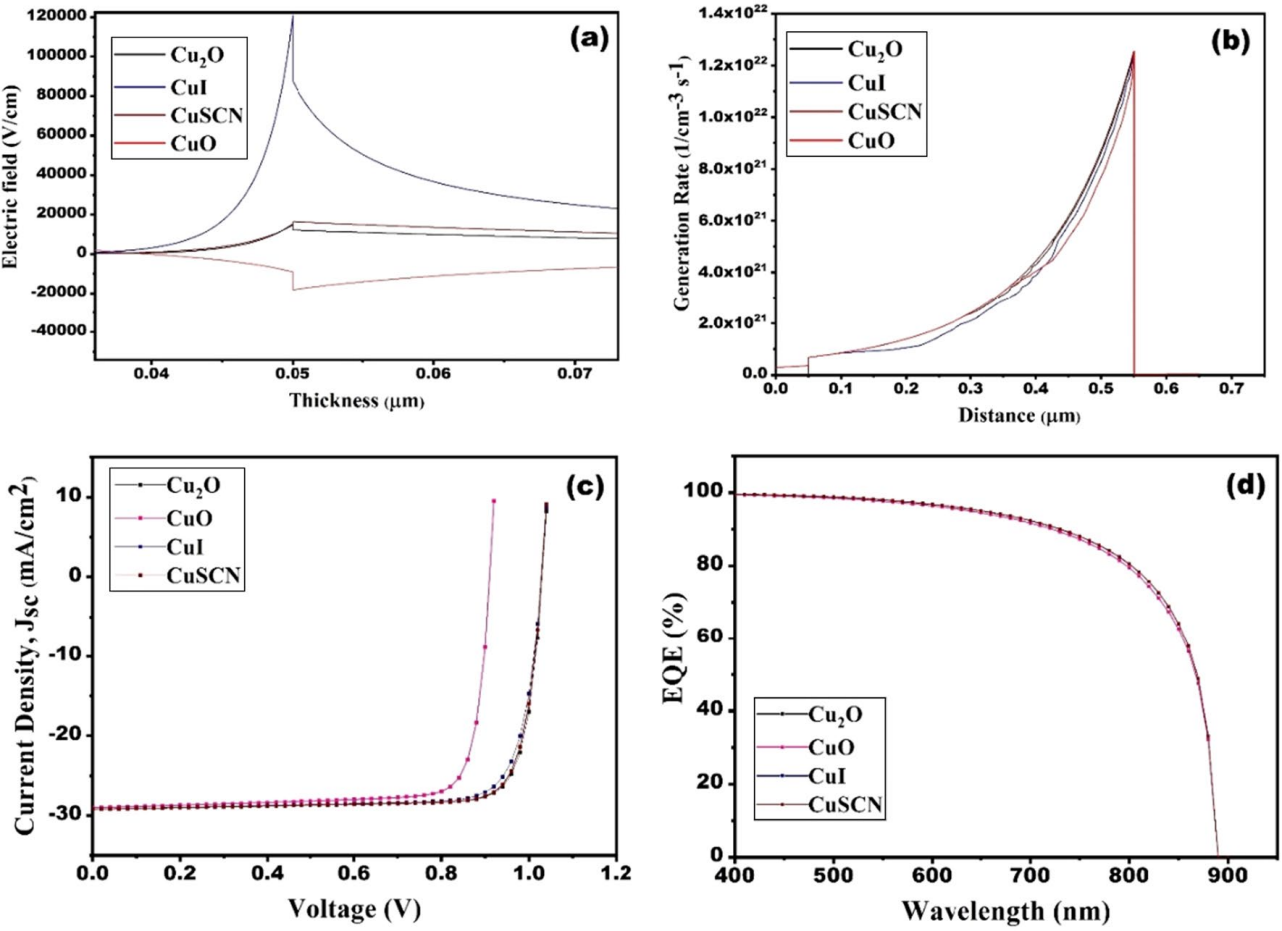
(**a**) Electric field, (**b**) generation rate (**c**) J–V curves and (**d**) EQE for different HTLs of solar cell.

From Table 5, it can be observed that Cu_2_O, CuI, and CuSCN have the same V_oc_ and J_sc_. Cu_2_O, on the other hand, has a quiet higher fill factor, and efficiency enhanced to 25.01%. The higher efficiency may be inferred from Fig. 4a and b, which display the generation rates across the device and the distribution of the electric field at the CZTS/HTLs interface, respectively. The generation rates for Cu_2_O and CuSCN are almost similar. However, the relatively reduced production rates for CuI and CuO result in low efficiency. Additionally, Fig. 4b shows the distribution of the electric field at the CZTS/HTLs contact. Despite its modest generation rates, the CuI exhibits the largest electric field, which further contributes to its excellent efficiency. However, since both Cu_2_O and CuSCN have essentially identical electric fields, less recombination occurs at the contact. CuO, on the other hand, has a lowered electric field that permits the migration of a minority electron towards the HTL, which promotes recombination and results in poor efficiency^37^. Figure 4c and d show the I–V and QE curves for different HTLs, which illustrate the fact that the QE responses of each HTL are similar. Thus, considering the efficiency, FF Cu_2_O has been taken as the HTL in further studies.

### Impact of HTL thickness on the performance of SC

**Figure 5:**
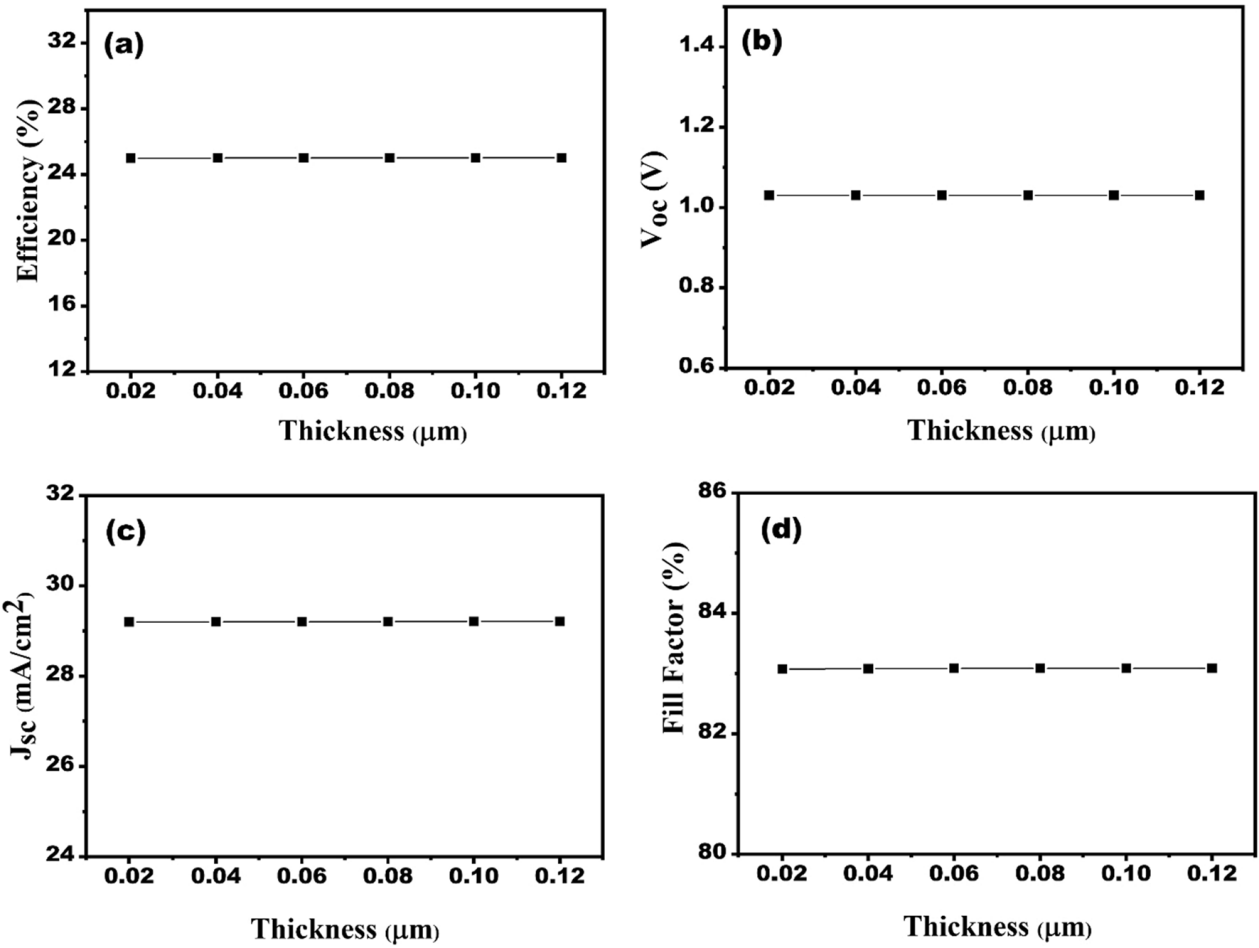
Effect of HTL thickness variation on the solar cell performances (**a**) efficiency, (**b**) V_oc_, (**c**) J_sc_ and (**d**) FF.

Proper thickness of HTL plays a crucial role in shaping high performance of the SC. The best HTL is the one that reduces electrical resistance and recombination setbacks, which further aids in boosting the SC's performance. Thickness of Cu_2_O material in designed SC structure is varied in the range of 0.02–0.12 μm. The thickness of Cu_2_O shows insignificant effect on the SC’s performance, as shown in Fig. 5a–d. However, Cu_2_O plays an active role in transporting the hole to the counter electrode owing to the making an energy cliff near the interface’s HTL/absorber. Thereby, the thickness of HTL is taken to be 50 nm for the optimized device structure.

### Impact of HTL doping density on the performance of SC

**Figure 6:**
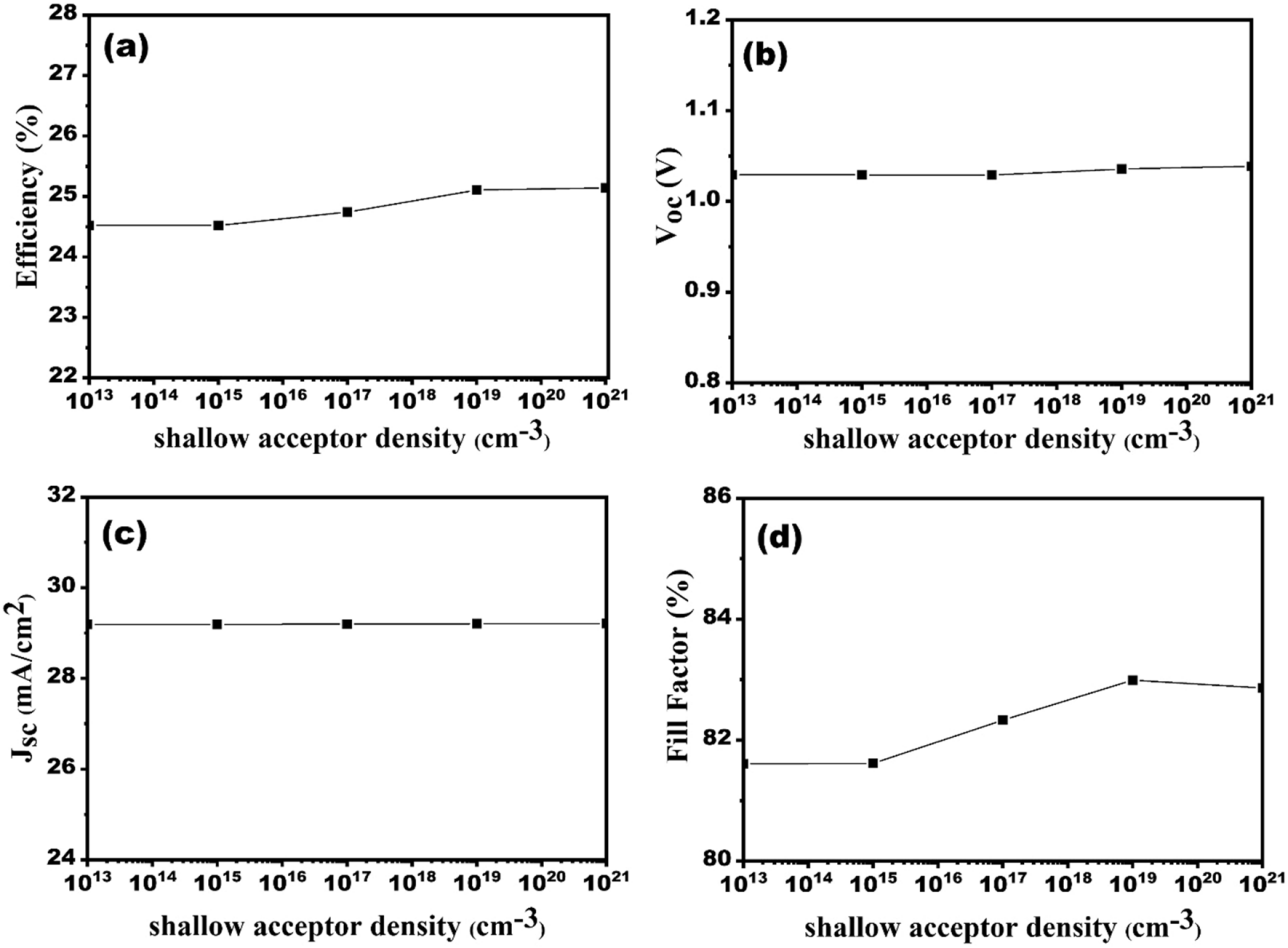
Effect of HTL acceptor doping density variation on the solar cell performances (**a**) efficiency, (**b**) V_oc_, (**c**) J_sc_ and (**d**) FF.

HTLs doping density controls SC performance by changing the electric field intensity at the HTL/absorber interface. An increase in electric field leads to a greater separation of electron–hole pairs and thus an increase in efficiency. Here, the acceptor density of HTL varies in between the range 10^13^–10^21^ per cm^3^. Figure 6a–d illustrate that increasing the acceptor concentration of Cu_2_O from 1 × 10^13^ per cm^3^ to 1 × 10^21^ per cm^3^ have no significant effect on the performance of SC. There are insignificant changes on *V*_*oc*_ and *J*_*sc*_ as represented in Figure 6b and c, respectively. However, we observed that there is a small change in efficiency from 24.14% to 25.14% (Fig. 6a) and FF from 81.61 to 82.86% (Fig. 6d). As we increase the doping concentration, charge carrier separation is increased and then the cell performances are also increased. The optimized result for acceptor concentration of Cu_2_O is considered 1 × 10^19^ per cm^3^.

### Impact of HTL electron affinity on the SC performance

**Figure 7:**
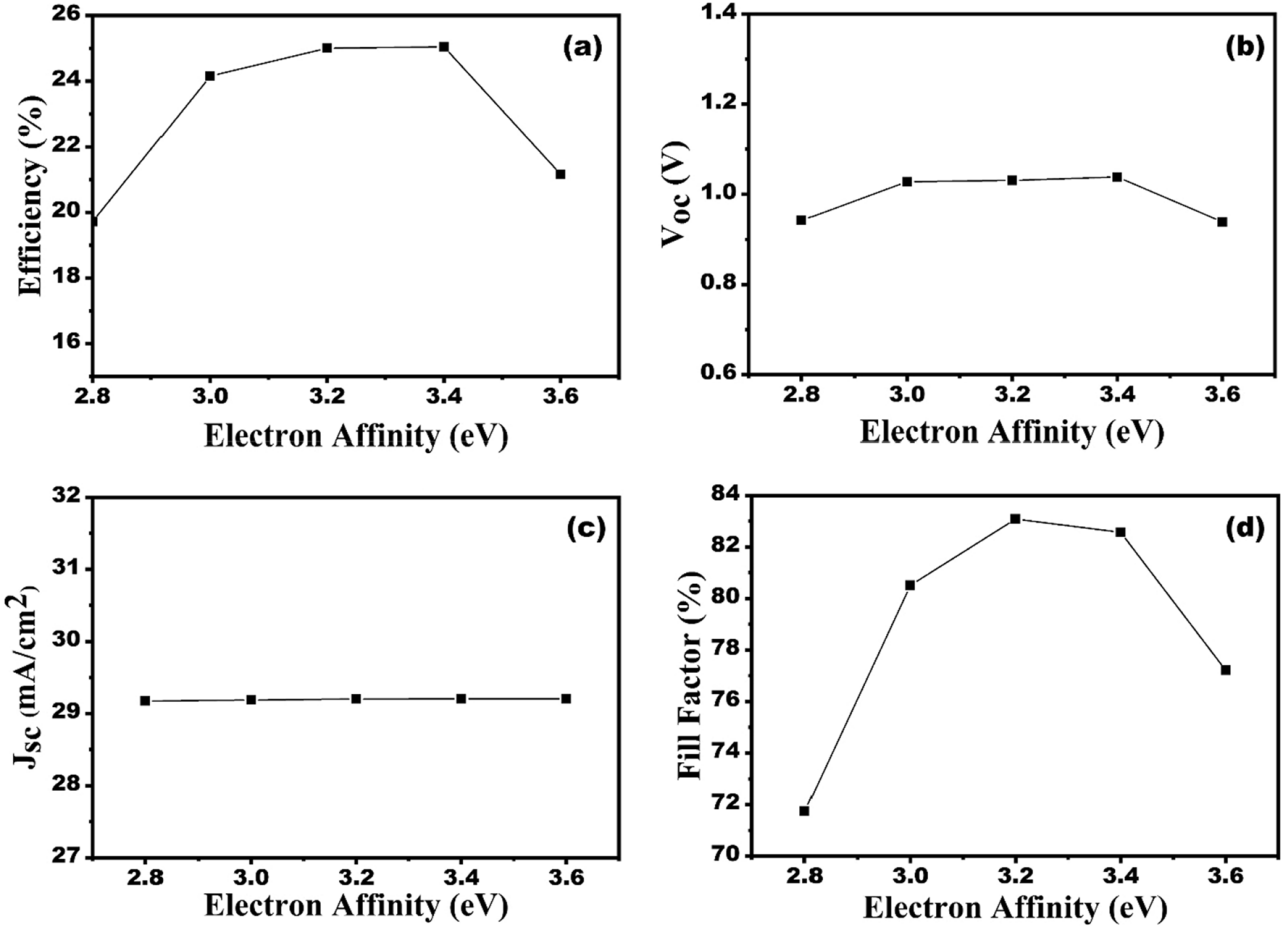
Effect of HTL electron affinity variation on the solar cell performances (**a**) efficiency, (**b**) V_oc_, (**c**) J_sc_ and (**d**) FF.

Electron affinity governs the contact conditions of the designed solar cell. A band offset is created when the electron affinities of the various layers differ, in the case of HTL, this is a valence band offset. In the present study, the impact of various HTL’s electron affinity is also studied on the SC performance parameters. The variation of Cu_2_O electron affinity has been performed considering that the bandgap of Cu_2_O be same (i.e. 2.17 eV). This will aid in selecting the best possible hole transport material with the proper band alignment for CZTS. Here, electron affinity is changes in between 2.8 and 3.4 eV, as shown in Fig. 7a–d. As the electron affinity increases from 2.8 to 3.4 eV, the V_oc_ usually increases and then begins to decrease after the electron affinity of 3.4 eV (Fig. 7b).

**Figure 8:**
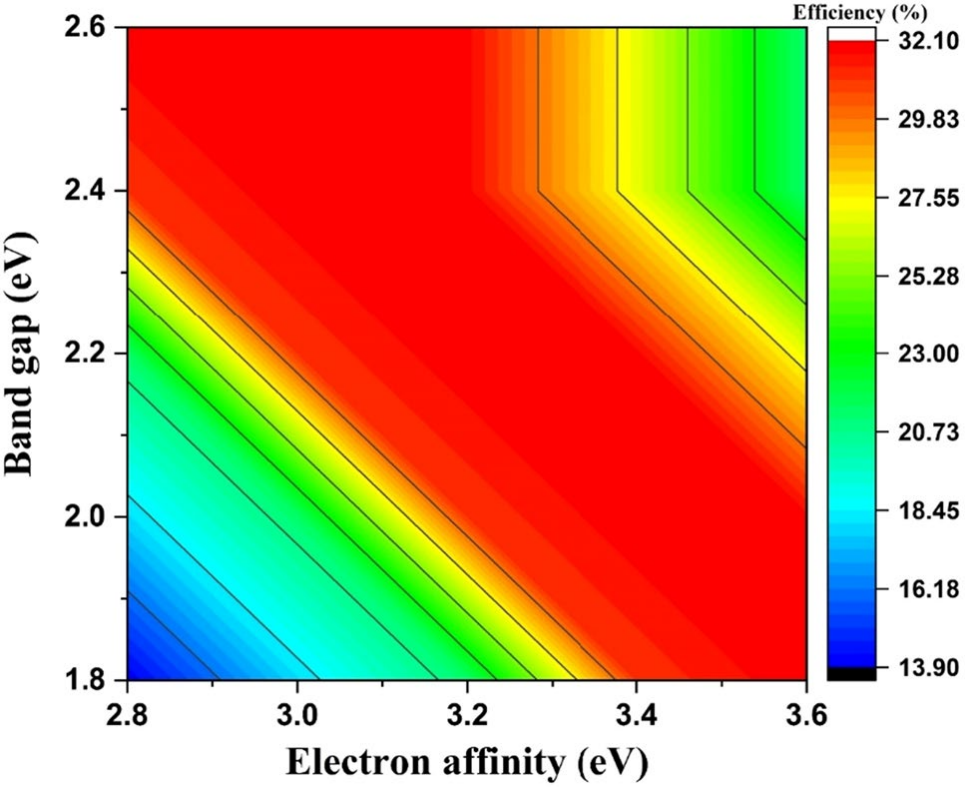
Contour diagram of efficiency as a function of bandgap and electron affinity of Cu_2_O for CZTS-based solar cell.

Figure 7c indicates that the J_sc_ is unaffected by changing electron affinity of HTL. Moreover, at electron affinities between 2.8 and 3.2 eV, the FF increases from 71 to 83% and then begins to drop after 3.2 eV (Fig. 7d). The same variation can be seen in Fig. 7a, where efficiency improves from 19.71 to 25.09% for affinities ranging from 2.8 to 3.4 eV before abruptly decreasing after 3.4 eV. The VBO will vary from − 0.53 to + 0.47 eV for electron affinities of 2.8 eV and 3.8 eV, respectively. At lower electron affinity the VBO becomes more negative, further forming the larger energy cliff. Further, the larger energy cliff will result in enhanced recombination probability at the HTL/CZTS interface^38^. Similarly, greater electron affinity will result in a higher positive VBO, creating a larger energy spike and increasing the chance of recombination. The increase in V_oc_, FF and efficiency with the HTL electron affinity of lower than 3.4 eV may be ascribed due to the minimization of recombination probability at the interface of HTL/CZTS, as manifested in Fig. 7. Additionally, as illustrated in Fig. 8, the impact of the HTL bandgap and electron affinity on efficiency is also examined. Therefore, it can be manifested from Fig. 8 that materials with bandgap over 2 eV and electron affinities in the range of 3.2–3.4 eV will be the most suitable HTL for this SC structure. Thus, to enhance the PV properties of the designed SC, the electron affinity of HTL is considered to be 3.2 eV.

### Impact of defect density of CZTS/ZnS and CZTS/Cu_2_O interface

**Figure 9:**
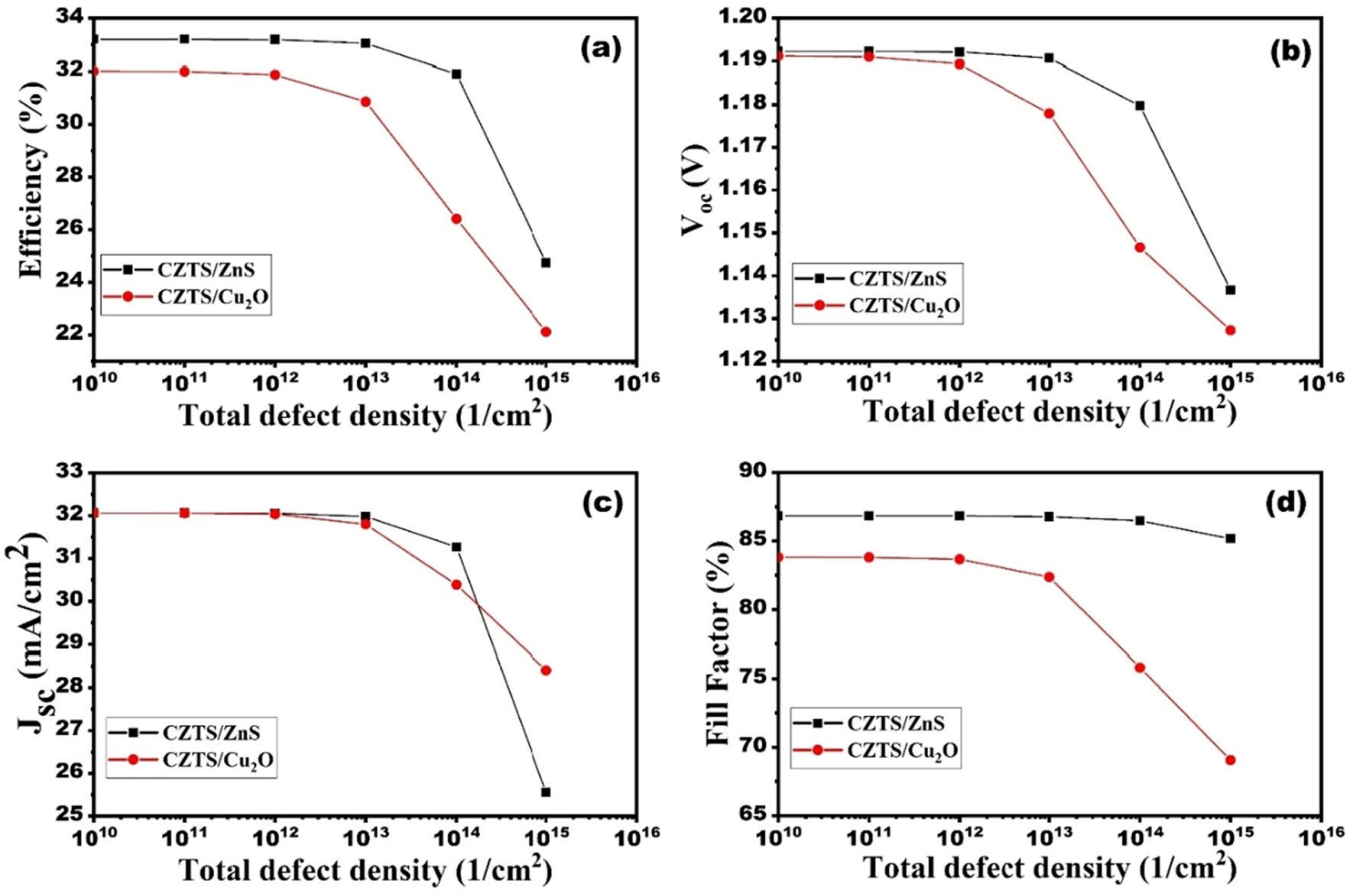
Variation in photovoltaic performance parameters against defect density at the CZTS/ZnS and CZTS/Cu_2_O interfaces for CZTS based solar cell.

Take into account that the incident photons must pass through the ETL/absorber interface before reaching the absorber layer. Exposure to extreme heat, light, oxygen, and humidity aggravates interface flaws, further impairing device performance. So, it's impossible to completely rule out the possibility of interface flaws^39,40^. Thus, to study the impact of CZTS/ZnS and CZTS/Cu_2_O interface defects, the defect density was varied from 10^10^ to 10^16^ cm^−2^. Figure 9a–d show the effect of defect density at the CZTS/ZnS and CZTS/Cu_2_O interfaces. It can be observed that the V_oc_, J_sc_, and FF remain almost constant up to 10^13^ cm^−2^ for both ETL and HTL. However, V_oc_, J_sc_, and FF degrade drastically after 10^13^ cm^−2^. This drastic decrease in solar cell parameters is due to increased recombination at higher defect densities. However, the results show that at the Cu_2_O/CZTS interface, the density of interface traps has a significantly greater impact on the overall performance than at the ZnS/CZTS interface. The reason for this is that the electric field developed at the interface is reduced, which promotes greater recombination. Accordingly, the interfacial defect density is taken to be 10^12^ cm^−2^ for both ZnS and Cu_2_O interfaces.

### Optimization of CZTS absorber layer

The thickness absorber material has a crucial effect on efficiency of the SC device. Light absorption and charge transfer are accomplished by the absorber layer. As a result, optimizing the absorber material is critical for improving SC performance. The change of doping density, thickness, and defect density is investigated here.

### Impact of CZTS absorber layer thickness on the performance of SC

**Figure 10:**
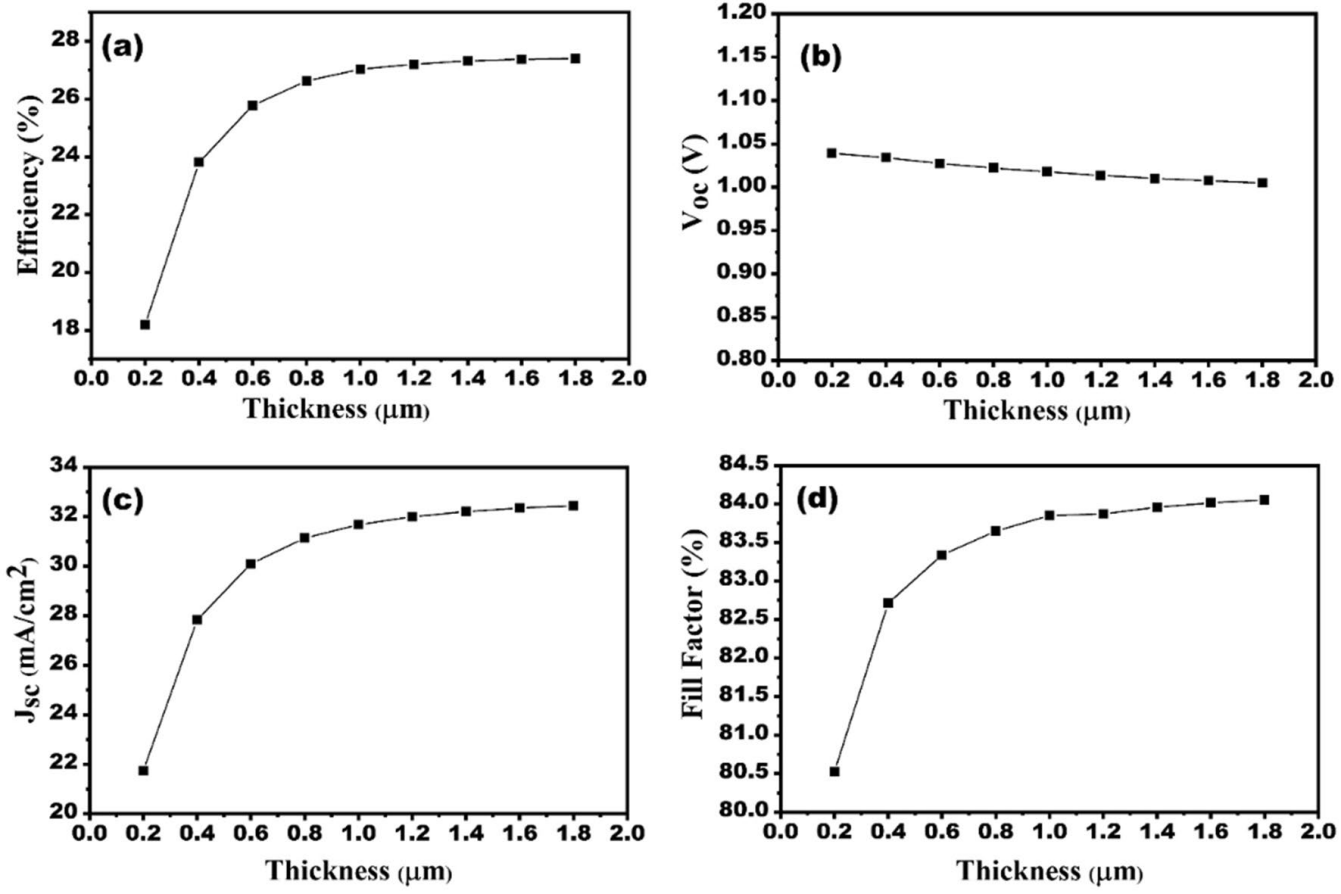
Effect of CZTS absorber layer thickness variation on the solar cell performances (**a**) efficiency, (**b**) V_oc_, (**c**) J_sc_ and (**d**) FF.

Figure 10a–d show the thickness of absorber material varies in the range between 0.2 and 1.8 µm. The rise in charge carrier recombination owing to the escalation in dark saturation current leads to a slight decrement in the V_oc_ (Fig. 10b). Efficiency V_oc_, and Jsc_,_ increases until 1.4 µm and then get saturated. As absorber material thickness reduces, the electrons and holes combine quickly, thereby increasing the rate of recombination. The rise in absorber thickness gives ample space for an increase in the number of photons to be absorbed, thus increasing the J_sc_, (Fig. 10c). Further, the FF also rises with increasing thickness. We observe a SC efficiency of 27.33% at the optimum absorber thickness (1.5 µm).

### Impact of absorber (CZTS) doping density on the SC performance

**Figure 11:**
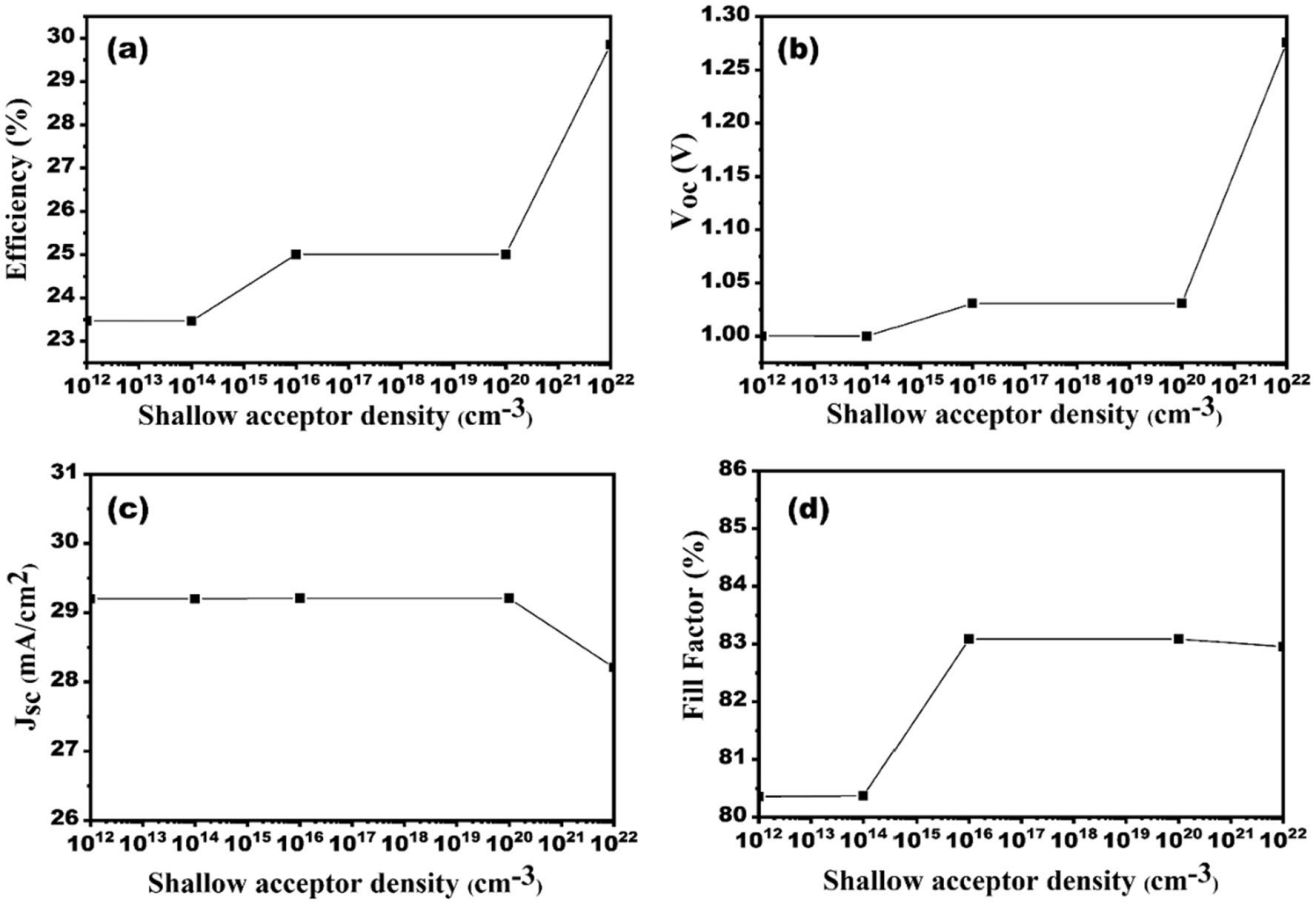
Effect of CZTS absorber layer doping density variation on the solar cell performances (**a**) efficiency, (**b)** V_oc_, (**c**) J_sc_ and (**d**) FF.

The acceptor concentration of absorber material varies in between 10^12^ and 10^22^ per cm^3^. It can be noticed from Fig. 11a–d that when the acceptor concentration of CZTS increases efficiency, V_oc_, and FF increases. However, J_sc_ remains constant and decreases when doping concentration goes beyond 1 × 10^20^ per cm^3^. This is because high doping concentration decreases the depletion region and charge carrier mobility which leads to increase in holes and electrons recombining and decreasing the chance of capturing electrons produced by photons. *V*_*oc*_ increases on increasing acceptor concentration. The device saturation current increases lead to increase in V_oc_. The efficiency increases from 23 to 29% as absorber material’s acceptor concentration are varies in between 10^12^ and 10^22^ per cm^3^. The optimum value of acceptor concentration for CZTS layer is taken as ~ 10^19^ per cm^3^. Columbic traps will be created by extremely high doping, which will increase recombination^41^.

### Impact of absorber layer (CZTS) defect density on the SC performance

**Figure 12:**
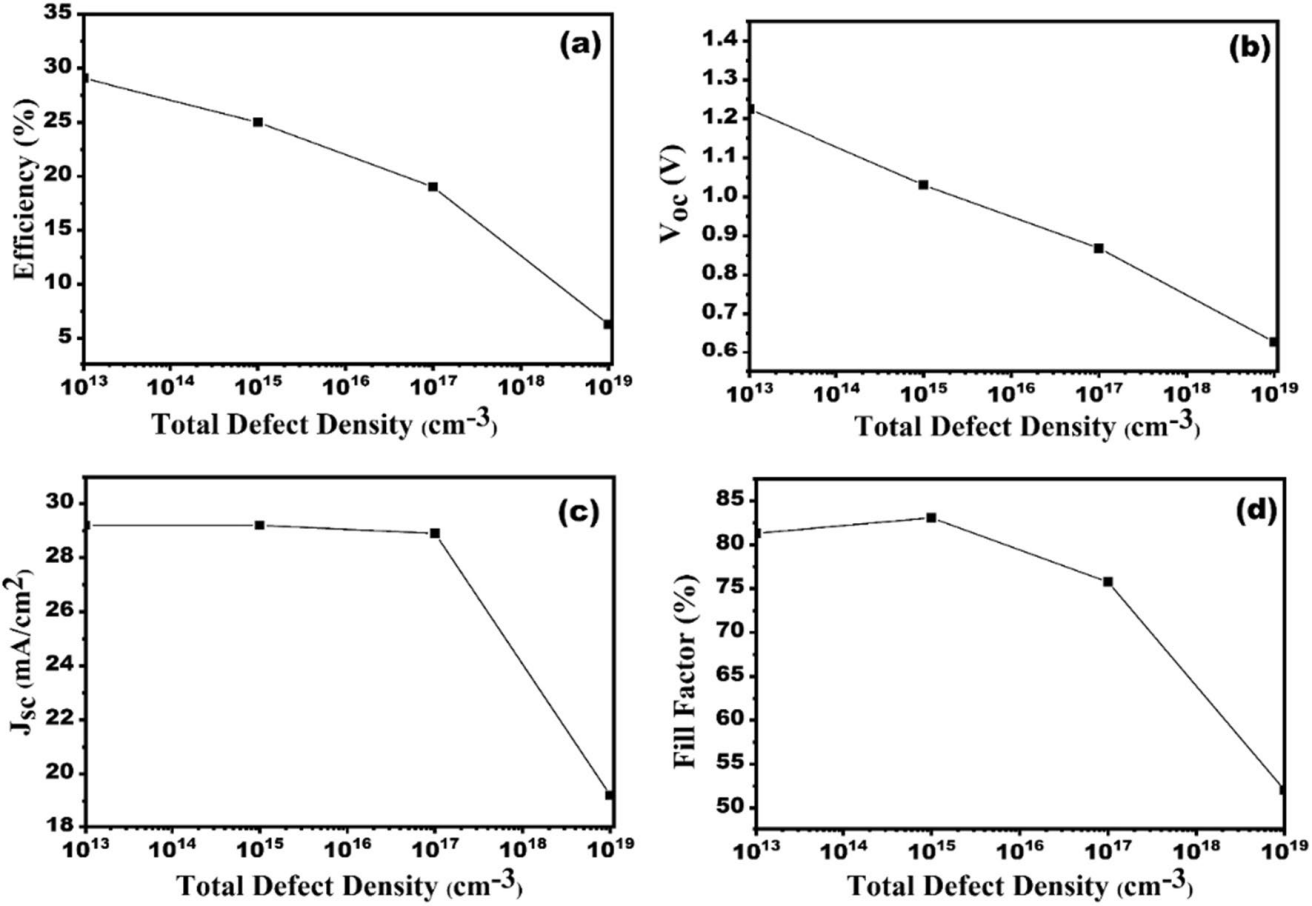
Effect of CZTS absorber layer defect density variation on the solar cell performances (**a**) efficiency, (**b**) V_oc_, (**c**) J_sc_ and (**d**) FF.

The impurities and defects are unavoidable during the manufacturing processes of SCs. Thus, to get a detailed overview of our designed CZTS-based SC, the defects are being introduced in absorber layers during simulation. The defects in absorber layer significantly affect the PCE of SCs. The Shockley–Read–Hall (SRH) recombination dominates due the existence of defects. The recombination increases owing to the existence of defects^42^. Higher defect density reduces the carrier lifetime and carrier diffusion length of the charge carriers generated due to absorption of photon^30,35,43^. Acceptor concentration of absorber materials varies in the range of 10^13^–10^19^ per cm^3^, can be seen in Fig. 12a–d. The V_oc_ falls sharply from 1.36 to 0.62 V, due to the increase in recombination at higher defect density, as revealed in Fig. 12b. In addition, Fig. 12c depicts that the J_sc_ remains constant up to 10^17^ cm^−3^ and shows a sudden decrease from 29.2 to 19.2 mA/cm^2^ at higher defect density. Consequently, the FF decreases from 82.44 to 52.06%. Besides, The SC's efficiency drops dramatically from 32.43 to 6.28% as a result of an increase in resistance and recombination, as shown ion Fig. 12a. Thus, the optimum defect concentration of 10^15^ per cm^3^ is set for the simulation.

### Impact of shunt and series resistances on the SC performance

**Figure 13:**
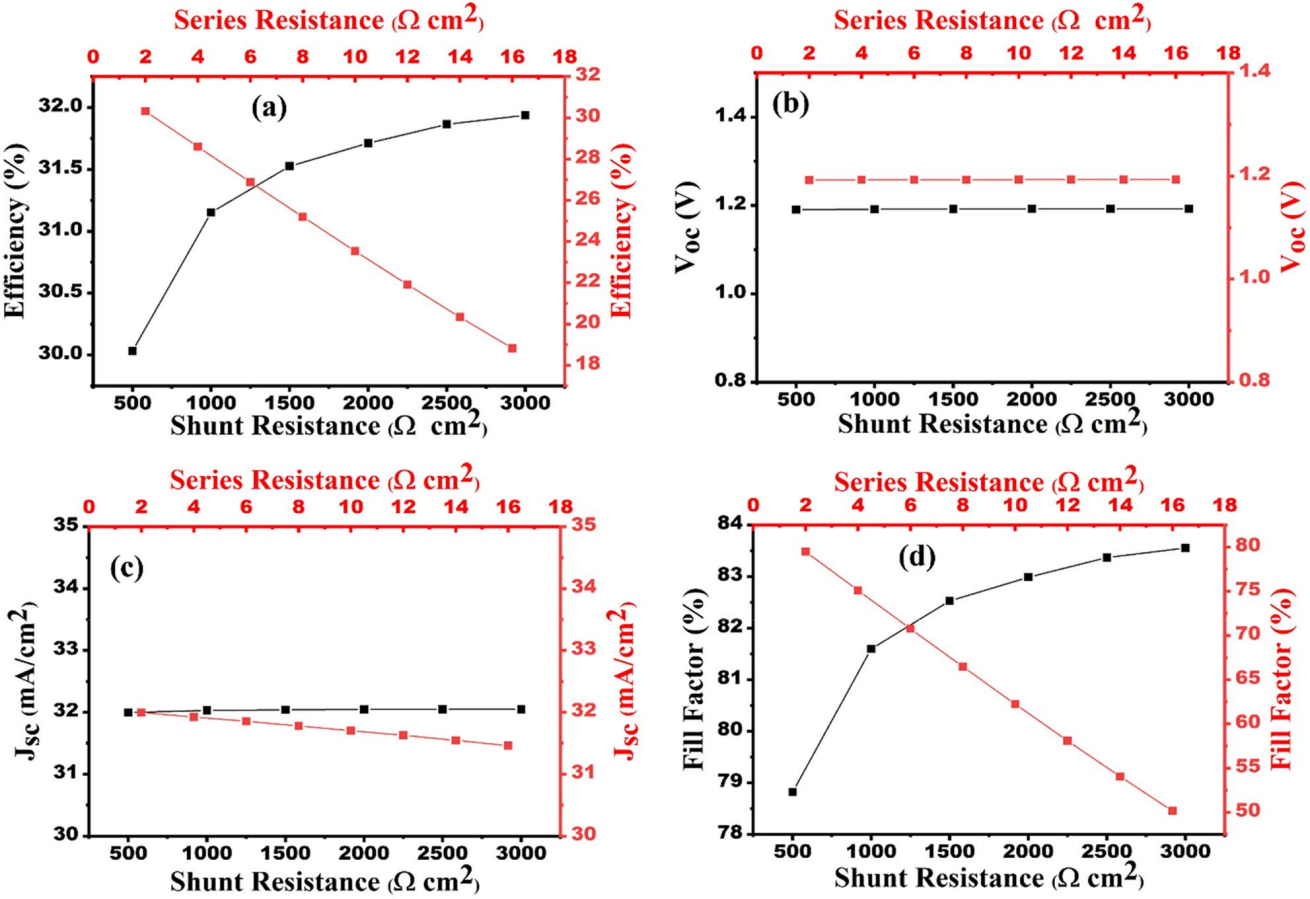
Effect of series and shunt resistance on the solar cell performances (**a**) efficiency, (**b**) V_oc_, (**c**) J_sc_ and (**d**) FF.

Resistivity is an intrinsic property of any material. Hence, the shunt and series resistances are considered during the optimization of designed SC. shunt (R_sh_) and Series (R_s_) resistances are attributed to contacts between layers and defects of materials, respectively^44–46^. Here, the Shunt and series resistances have been varied from 2 to 16 Ω cm^2^ and 500 to 3000 Ω cm^2^, respectively, as shown in Fig. 13a–d. It can be observed from Fig. 13b and c that there is a negligible effect of shunt and series resistance on J_sc_ and V_oc_, respectively. Furthermore, the increasing series resistance advances the decrease in FF and efficiency from 79.49 to 50.15% and 30.32 to 18.82%, respectively. On the contrary, the increasing shunt resistance gives rise to FF and efficiency, which increase from 78.82 to 83.55% and 30.03 to 31.92%, respectively. This may be ascribed to the decline the value of leakage current in the designed SC. Thus, the optimum value of series and shunt resistance is manifested in the range of 0–4 Ω cm^2^ and 2500–3000 Ω cm^2^, correspondingly.

### Impact of operating temperature on the device performance

**Figure 14:**
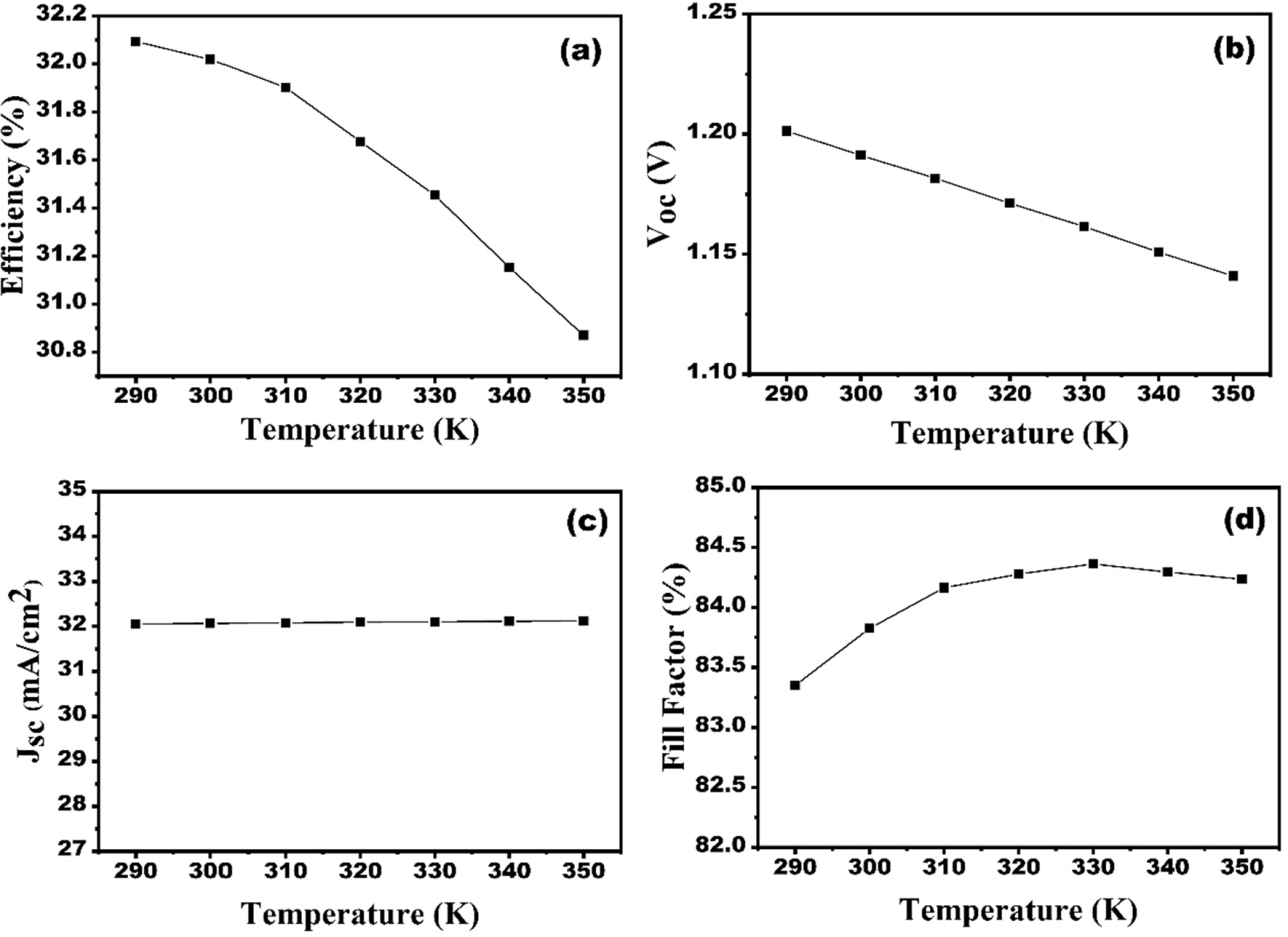
Effect of  operating temperature on the solar cell performances (**a**) efficiency, (**b**) V_oc_, (**c**) J_sc_ and (**d**) FF.

The operating temperature of an SC device affects its performance. The hole, electron mobilities and material carrier concentration are impressed with higher operating temperature of the PV device, which leads to lower efficiency^47^. The operating temperature is measured in between of 290–350 K and the resulting outcomes are shown in Fig. 14a–d. Figure 14b indicates that the increase in operating temperature leads to decline the V_oc_. The V_oc_ decreases because the temperature’s rise leads to a shrinkage in the bandgap of the absorber^36^. The J_sc_ is unaffected by varying temperatures. Figure 14d shows that the FF increases with increasing temperature, indicating that there is an increment in the power output of the device. However, there is a minor drop in efficiency (Fig. 14a) from 32.09 to 30.88%. Because of the high temperature, the holes and electrons combine faster before reaching the depletion area, leading to a decrease in device efficiency^48^. Therefore, the designed CZTS-based SC is highly stable at higher temperatures. Thus, the optimum operating temperature is chosen to be 300 K.

### Optimized result and I–V curve

After considering all the optimization parameters, the optimized values for different material parameters are tabulated in Table 6.

**Table 6:** Optimized values for various layers of CZTS-based SC.

Optimized PV parameter	CZTS	Cu_2_O
Thickness (µm)	1.5	0.05
Acceptor doping density (cm^−3^)	1 × 10^19^	1 × 10^19^
Defect density (cm^−3^)	1 × 10^15^	–
Interface defect density (cm^−2^)	CZTS/Cu_2_O = 10^12^	CZTS/ZnS = 10^12^
Electron affinity (eV)	–	3.2
Series and shunt resistance (Ω cm^2^)	R_s_ = 0–4, R_sh_ = 2500–3000	
Temperature	300 K	

**Figure 15:**
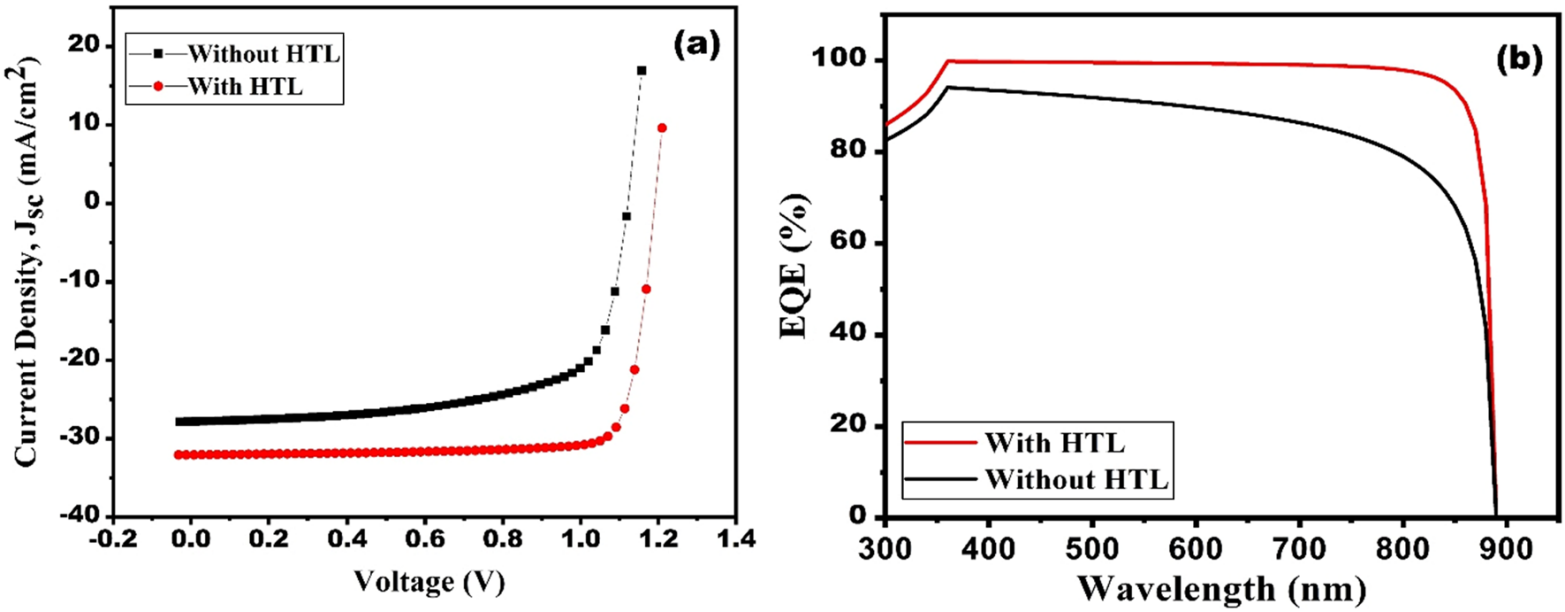
The comparative (**a**) J–V curve and (**b**) EQE for designed solar cell with and without HTL.

In addition to that the SC parameters with and without HTL is also shown in Table 6. The role of HTL (Cu_2_O) in the proposed structure of CZTS based SC. Figure 15a represents the J–V and QE curve of CZTS-based SC with HTL (FTO/ZnS/CZTS/Cu_2_O/Au) and without HTL (FTO/ZnS/CZTS/Au). It can be manifested from Table 7 that the FF and efficiency are enhanced after the introduction of Cu_2_O as HTL. At optimized device parameters the efficiency with and without HTL is found to be 31.86% and 21.17%, respectively. This is because of the interfacial recombination of charge carriers between CZTS and back contact metal (gold)^37^. Figure 15b shows a sharp decrease in QE response beyond 885 nm, confirming the band gap of CZTS, which is 1.4 eV. It is also evident that the QE response of SC using Cu_2_O as HTL is higher than the QE response of solar cells without HTL. This difference in QE response can be attributed to the generation of a back surface field after the incorporation of HTL^38,49^. CZTS can act both as absorber and p-type layer. After adding another p-type layer (Cu_2_O) the performance of SC improves. So, Cu_2_O as HTL is required for better performance of the proposed SC.

**Table 7:** Performance parameters of CZTS-based both without HTL and with HTL.

Structure (FTO/ZnS/CZTS/Cu-based HTL/Au)	V_oc_ (V)	J_sc_ (mA/cm^2^)	Fill factor (%)	Efficiency (%)
Without HTL	1.12	27.84	67.45	21.06
With HTL (Cu_2_O)	1.19	32.05	83.37	31.86

### Capacitance–voltage (C–V) for the optimized structure

Apart from the J–V curve, C–V is one of the most important parameters to study to get a better insight of the built-in potential (*V*_*bi*_) of the proposed SC heterostructure. In addition to that the doping density can also be calculated using the C-V curve, by using the equations^43^

2 $$\frac{1}{{C}^{2}}= \frac{2}{q{N}_{a}{\varepsilon }_{0}{\varepsilon }_{s}{A}^{2}}({V}_{bi}- V)$$

3 $${N}_{a}= \frac{2}{q{\varepsilon }_{0}{\varepsilon }_{s}{A}^{2}\left[\frac{d}{dv}\frac{1}{{C}^{2}}\right]}$$

here, *ε*_*0*_is permittivity of free space (8.85 × 10^–14^ F cm^−1^), *N*_*a*_ and *q* are doping density (in per cm^3^) and charge on electron (1.6 × 10^−19^C). *ε*_*s*_, *A*, *C* and *V* are relative dielectric constant (refer Table 2), area of SC (in square-cm), capacitance, and applied potential respectively.

**Figure 16:**
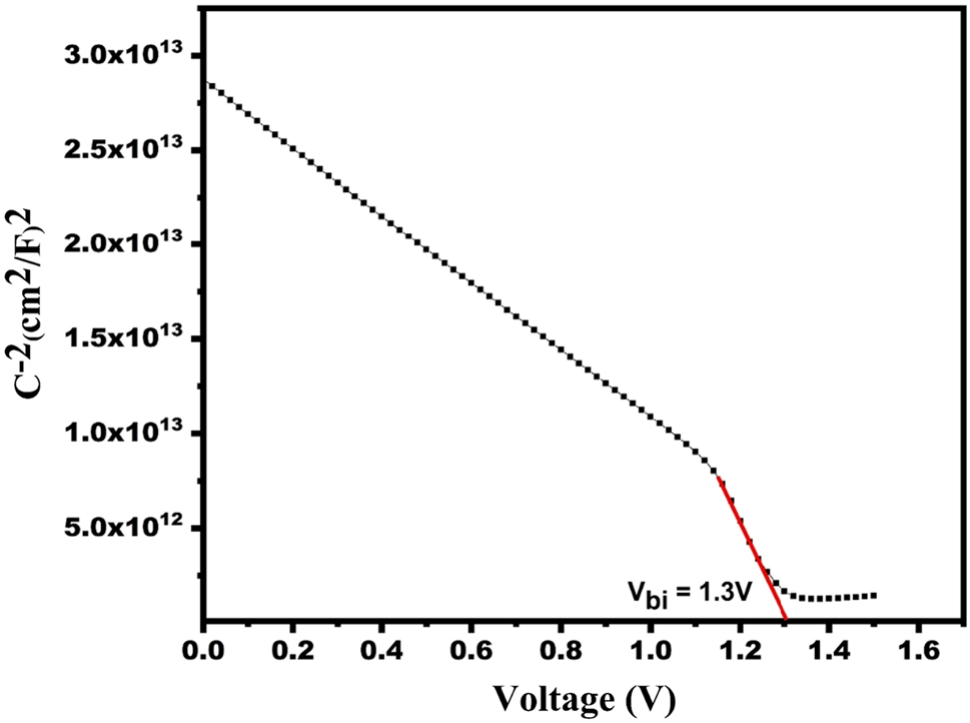
The C–V curve for designed solar cell.

Figure 16 indicates the graph between 1/*C*^2^ vs. *V*. We have calculated built-in potential (*V*_*bi*_) from the intercept of the graph. The V_bi_ is found to be 1.3 V for the CZTS-based SC. The improved efficiency of the SC is owing to large V_bi_, which enhances the separation of charge carriers. Further, the doping density (N_A_) is calculated by using Eq. (2) and found the value to be approx. 1 × 10^19^ cm^−3^. The calculated value of doping density is nearly the optimized doping density of CZTS material (Table 2).

## Conclusions

The suggested structure, which consists of FTO/ZnS/CZTS/Cu_2_O/Au, is simulated using the SCAPS-1D programme. It has been noticed that the SC's efficiency may improve by selecting the optimum value thickness and charge carrier concentration for absorber material and HTL. The optimized thickness of absorber, HTL is 1.5 µm and 0.05 µm, respectively, and optimized doping concentration is 1.0 × 10^19^ per cm^3^ for both absorber and HTL. We achieved excellent results such as efficiency ~ 31.86%, J_sc_ ~ 32.05 mA cm^**−**2^, V_oc_ ~ 1.19 V and the FF ~ 83.37%. The calculated doping density ~ 1 × 10^19^ cm^−3^ is similar to the optimized doping density of CZTS material. We observed built-in potential 1.3 V for the CZTS-based SC. The outcomes showed that the thin layer of ZnS may be used in the creation of SCs and offers a potential alternative for the commonly employed hazardous CdS as ETL. Sustainable development may be facilitated by the use of low-cost, non-toxic, and plentiful CZTS.

## Data Availability

Data and materials for this study are available and will be provided to the journal upon request.
